# Porcine reproductive and respiratory syndrome virus (PRRSV) infection spreads by cell-to-cell transfer in cultured MARC-145 cells, is dependent on an intact cytoskeleton, and is suppressed by drug-targeting of cell permissiveness to virus infection

**DOI:** 10.1186/1743-422X-3-90

**Published:** 2006-11-02

**Authors:** William A Cafruny, Richard G Duman, Grace HW Wong, Suleman Said, Pam Ward-Demo, Raymond RR Rowland, Eric A Nelson

**Affiliations:** 1 Division of Basic Biomedical Science, Sanford School ofMedicine, University of South Dakota, Vermillion, SD 57069, USA; 2 Division of Basic Biomedical Sciences, Sanford School ofMedicine, University of South Dakota, Vermillion, SD 57069, USA; 3 Actokine Therapeutics, 12 Middlesex Rd. Chestnut Hill, MA02467, USA; 4 Division of Basic Biomedical Sciences, Sanford School ofMedicine, University of South Dakota, Vermillion, SD 57069, USA; 5 Sanford School of Medicine, University of South Dakota, Vermillion, SD 57069, USA; 6 Department of Diagnostic Medicine and Pathobiology, KansasState University, Manhattan, KS 66506, USA; 7 Department of Veterinary Science, South Dakota State University, Brookings, SD 57007, USA

## Abstract

**Background:**

Porcine reproductive and respiratory syndrome virus (PRRSV) is the etiologic agent of PRRS, causing widespread chronic infections which are largely uncontrolled by currently available vaccines or other antiviral measures. Cultured monkey kidney (MARC-145) cells provide an important tool for the study of PRRSV replication. For the present study, flow cytometric and fluorescence antibody (FA) analyses of PRRSV infection of cultured MARC-145 cells were carried out in experiments designed to clarify viral dynamics and the mechanism of viral spread. The roles of viral permissiveness and the cytoskeleton in PRRSV infection and transmission were examined in conjunction with antiviral and cytotoxic drugs.

**Results:**

Flow cytometric and FA analyses of PRRSV antigen expression revealed distinct primary and secondary phases of MARC-145 cell infection. PRRSV antigen was randomly expressed in a few percent of cells during the primary phase of infection (up to about 20–22 h p.i.), but the logarithmic infection phase (days 2–3 p.i.), was characterized by secondary spread to clusters of infected cells. The formation of secondary clusters of PRRSV-infected cells preceded the development of CPE in MARC-145 cells, and both primary and secondary PRRSV infection were inhibited by colchicine and cytochalasin D, demonstrating a critical role of the cytoskeleton in viral permissiveness as well as cell-to-cell transmission from a subpopulation of cells permissive for free virus to secondary targets. Cellular expression of actin also appeared to correlate with PRRSV resistance, suggesting a second role of the actin cytoskeleton as a potential barrier to cell-to-cell transmission. PRRSV infection and cell-to-cell transmission were efficiently suppressed by interferon-γ (IFN-γ), as well as the more-potent experimental antiviral agent AK-2.

**Conclusion:**

The results demonstrate two distinct mechanisms of PRRSV infection: primary infection of a relatively small subpopulation of innately PRRSV-permissive cells, and secondary cell-to-cell transmission to contiguous cells which appear non-permissive to free virus. The results also indicate that an intact cytoskeleton is critical for PRRSV infection, and that viral permissiveness is a highly efficient drug target to control PRRSV infection. The data from this experimental system have important implications for the mechanisms of PRRSV persistence and pathology, as well as for a better understanding of arterivirus regulation.

## Background

Porcine reproductive and respiratory syndrome virus (PRRSV) is an arterivirus which is the etiologic agent of PRRS, a disease of epidemic proportions in swine [1-3]. PRRSV is macrophage-tropic *in vivo*, where it establishes a chronic infection, and the virus replicates in primary pig macrophages *in vitro *[4-6]. PRRSV infection has been extensively studied in MARC-145 cells, a PRRSV-permissive monkey kidney cell line [7,8]. Previous studies have established that PRRSV replication in cultured MARC-145 cells follows a complex time-course, with PRRSV antigens becoming detectable by immunofluorescence analysis between about 10–20 h p.i., and emergence of foci of damage (cytopathic effect; CPE) usually over the next 3–4 days [7,8].

The fate of PRRSV-infected MARC-145 cell cultures may include death of some cells by modified apoptosis [9] or necrosis [10], as well as establishment of chronic PRRSV infection (Cafruny & Rowland, unpublished). Thus, clarifying the behavior of PRRSV in MARC-145 cells is significant to progress in developing anti-viral strategies.

Previous studies have suggested that initial defenses against PRRSV are comprised of innate lung and alveolar macrophage responses [6]; subsequently, both Th1 and Th2 responses are induced in the respiratory tracts of PRRSV-infected pigs [11]. PRRSV infection of pigs is associated with activation of several cytokines including interferon-γ [IFN-γ; [12,13]], which has PRRSV-inhibitory activity *in vitro *[14]. However, the IFN-γ response to PRRSV may be inhibited or delayed by some unknown factors during PRRSV infection or vaccination [15,16], and ultimately a poorly-neutralizing Th2-dependent response seems to result in many pigs. Combined, the characteristics of these host responses may facilitate viral persistence [15,16].

The interaction of PRRSV with host cytokines is not well understood, but this area of study is a potential key to understanding host mechanisms during infection. Cytokines have not yet been exploited to control PRRSV infection *in vivo*, but their potential to regulate PRRSV infection in certain experimental systems provides a rationale for PRRSV discovery research, and anti-PRRSV agents may be important tools for future drug development.

The viral dynamics of another arterivirus, lactate dehydrogenase-elevating virus (LDV), are dominated by regulation of the LDV-permissive state; only a small fraction of mouse macrophages are susceptible to LDV infection, leading to an avirulent chronic infection in most mice which is maintained through development of newly-permissive cells [17]. Viral permissiveness is a logical but poorly exploited target for antiviral drugs [18-20], and the present study utilized two antiviral agents which target permissiveness (IFN-γ and an experimental antiviral known as AK-2), as well as cytoskeleton disruptors, to probe checkpoints in PRRSV replication.

Initially, our goal was to better characterize the dynamics of PRRSV replication in MARC-145 cells. Using flow cytometry and fluorescence microscopy, we demonstrated logarithmic growth of PRRSV in MARC-145 cells, culminating over a period of 3–4 days in the death of most cells. Secondary spread of PRRSV infection was observed to be via cell-to-cell transmission, as demonstrated by emergence of clusters of PRRSV-infected cells in confluent monolayers of MARC-145 cells, which preceded PRRSV-induced CPE, were inhibited by colchicine and cytochalasin D, and correlated with reduced actin expression. PRRSV replication was sensitive to IFN-γ as well as AK-2, which was a relatively more potent PRRSV inhibitor and capable of suppressing both primary and secondary PRRSV infection. The results of this study demonstrate cell-to-cell spread of PRRSV in cultured MARC-145 cells, the dependence of PRRSV infection and transmission on an intact cytoskeleton, and highlight the role of the PRRSV-permissive state as a critical drug target, with important implications for future therapeutic and preventive strategies.

## Results

### PRRSV replication dynamics in MARC-145 cells

**Figure 1:**
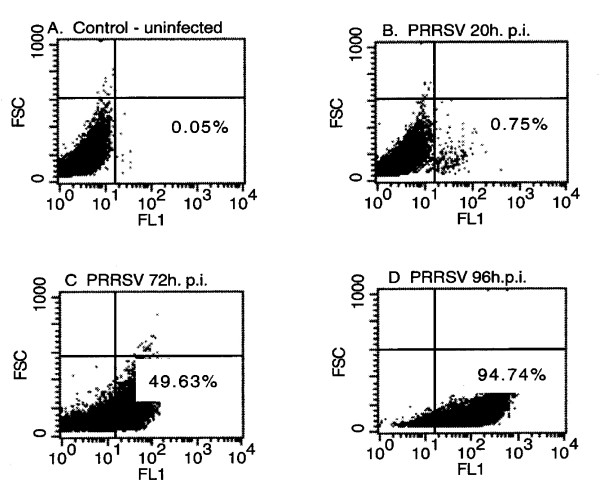
**PRRSV replication dynamics in MARC-145 cells by flow cytometry**. A. Control (uninfected) cells; B. 20 h p.i. with PRRSV; C. 72 h p.i. with PRRSV; D. 96 h p.i. with PRRSV. The percentage of PRRSV antigen-positive cells is shown in the lower right quadrant for each graph.

**Figure 2:**
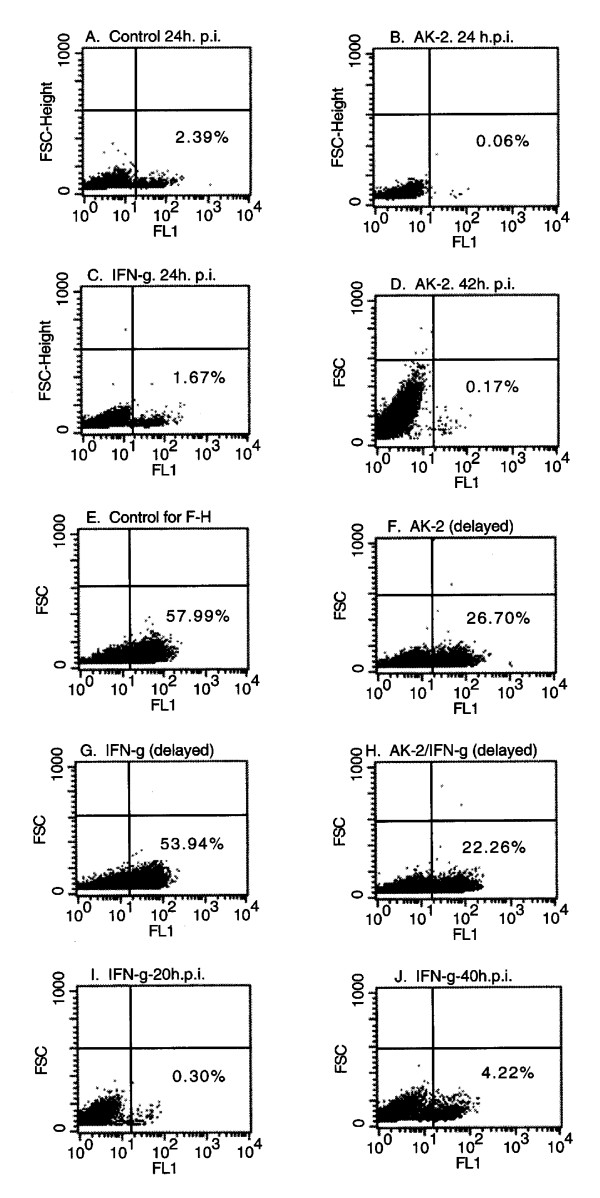
**Effects of AK-2 and IFN-γ on PRRSV replication in MARC-145 cells as determined by flow cytometry**. A. Control 24 h p.i. with PRRSV; B. AK-2 pretreatment, 24 h p.i. with PRRSV; C. IFN-γ pretreatment, 24 h p.i. with PRRSV; D. AK-2 pretreatment, 42 h p.i. with PRRSV; E. Control 46 h p.i. with PRRSV; F. AK-2 started at 18 h p.i. (delayed); G. IFN-γ started at 18 h p.i. (delayed); H. AK-2 and IFN-γ in combination, started at 18 h p.i.; I & J. IFN-γ-pretreatment effects on primary (I) and secondary (J) response PRRSV antigen detection. The percentage of PRRSV antigen-positive cells is shown in the lower right quadrant for each graph.

When PRRSV replication was assessed in MARC-145 cells at 20–22 h p.i., only a small proportion of cells expressed PRRSV antigen, as determined by FA (<5% in > 10 manual experiments counting fluorescent-positive cells under the microscope; Figure 1 by flow cytometry). However, between 42–72 h p.i. the percent of PRRSV-positive cells increased rapidly as determined by flow cytometric analyses, up to a maximum of about 95% by 96 h p.i. (Figures 1 &2). Low permissiveness of MARC-145 cells to primary (less than about 22 h p.i.) PRRSV infection was not due to insufficient M.O.I., since inoculation of cultures with about 100-times the standard dose resulted in a maximum 5.5% incidence of PRRSV-positive cells at 22 h p.i. by flow cytometry (data not shown). Propidium iodide staining followed by flow cytometry showed that PRRSV inoculated cells underwent major changes in cell cycle parameters (e.g. drop in G1 and G2; increased debris and aggregates) between 72–96 h p.i., consistent with cell damage and the spread of microscopic foci (CPE) typically observed throughout the cultures (data not shown).

**Figure 3:**
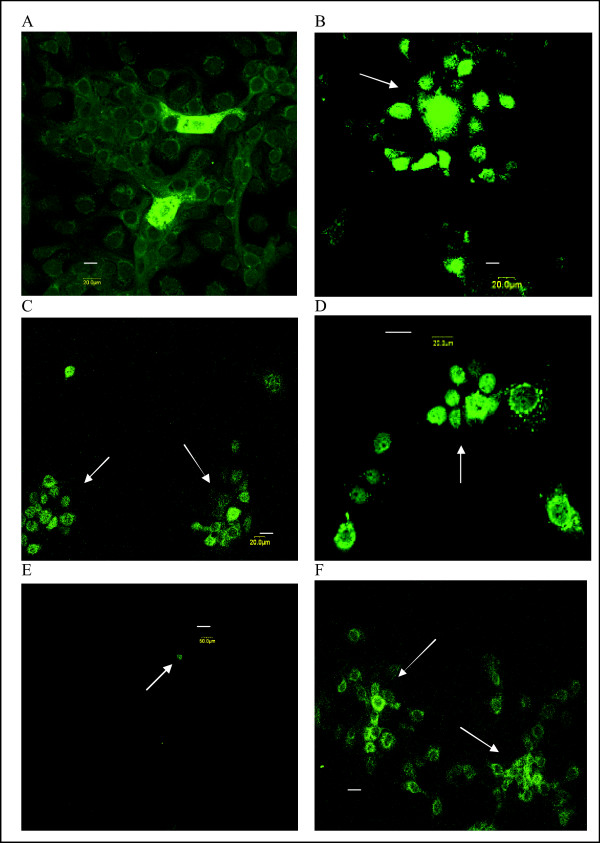
**Confocal microscopy of FA-stained MARC-145 cells during PRRSV infection**. A. 24 h p.i. with PRRSV, 20 μm.; B-C. Secondary cluster formation (arrows) 42–46 h p.i. with PRRSV, 20 μm; D. Secondary cluster formation (arrow) 72 h p.i. with PRRSV, 20 μm.; E. AK-2 pretreatment, 42 h p.i.; arrow indicates a single PRRSV-positive cell, 50 μm; F. AK-2 post-treatment, 46 h p.i., arrows indicate several PRRSV-positive clusters, 20 μm. (Length of scale bar is indicated for each panel)

FA analyses by confocal fluorescence microscopy, of dozens of independent PRRSV-infected confluent cultures, revealed that the primary (< 22 h p.i.) phase of PRRSV infection was characterized by random targeting of PRRSV-permissive single cells (Figure 3A; see also Figure 5B for another example), representing only a few percent of the total cells, and consistent with the quantitative analyses by flow cytometry.

**Figure 4:**
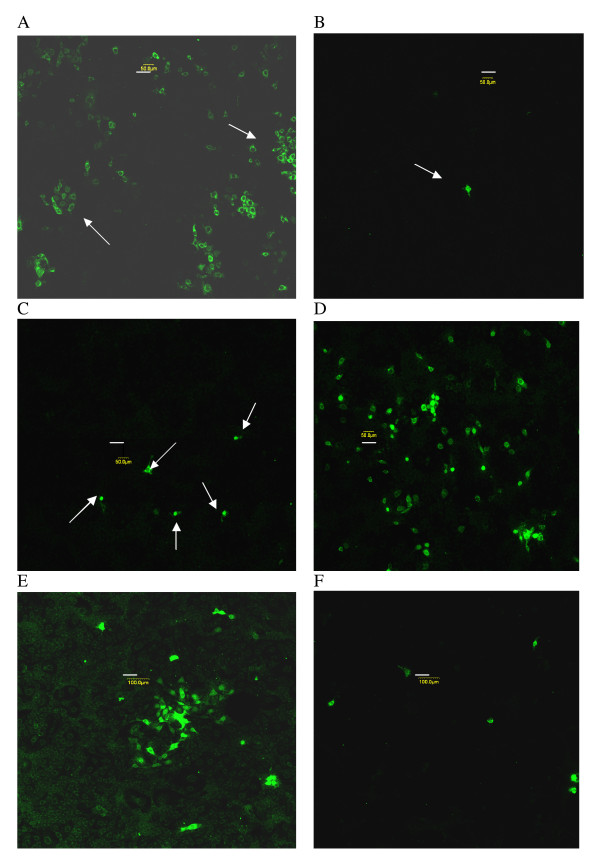
**Confocal microscopy of FA-stained MARC-145 cells during PRRSV infection**. A. IFN-γ pretreatment, 46 h p.i., arrows indicate several PRRSV-positive clusters, 50 μm; B. AK-2 + IFN-γ post-treatment, arrow indicates a single PRRSV-positive cell, 46 h p.i., 50 μm; C. AK-2 pretreatment, 42 h p.i., occasional PRRSV-positive cells (arrows), 50 μm; D. IFN-γ pretreatment, 50 μm; E. 45 h p.i. with PRRSV, 100 μm; F. Simultaneous AK-2 treatment and PRRSV infection, 45 h p.i., 100 μm. (Length of scale bar is indicated for each panel)

**Figure 5:**
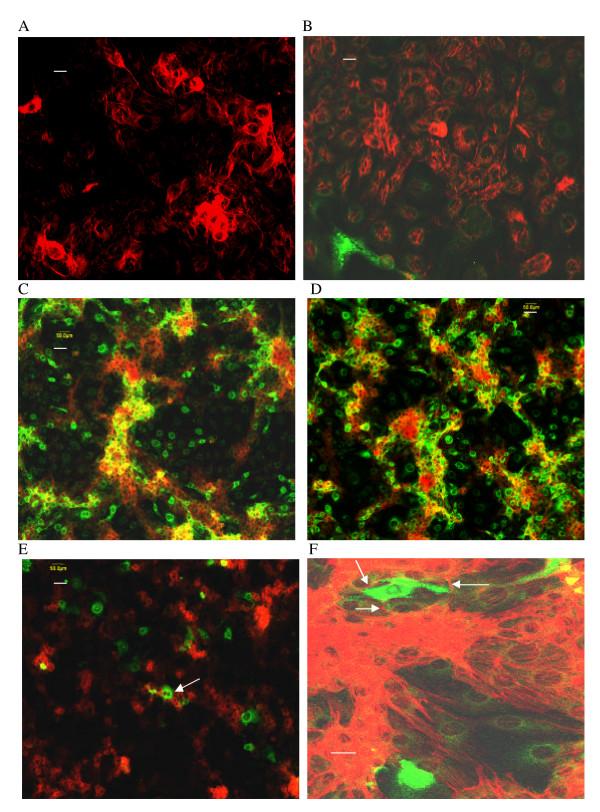
**Two-color fluorescence detection of actin (red) and PRRSV antigen (green) in MARC-145 cells**. A. Uninfected control, 20 μm; B.18 h p.i. with PRRSV, 20 μm; C. Control at 42 h p.i. with PRRSV, 50 μm; D. Drug vehicle control at 42 h p.i. with PRRSV, 50 μm. E. Colchicine-treated, 5 μM, 42 h p.i. (arrow indicates PRRSV-positive doublet; see Figure 6, A, C and D for higher magnification), 50 μm; F. AK-2 treated, 250 μm; (Length of scale bar is indicated for each panel)

### Formation of PRRSV-infected cell clusters (secondary infection)

**Figure 6:**
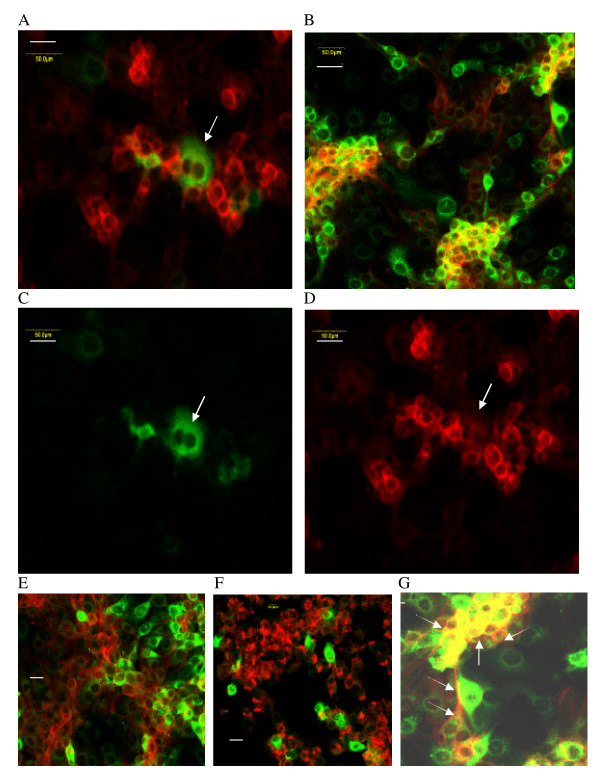
**Two-color fluorescence detection of actin (red) and PRRSV antigen (green) in MARC-145 cells**. A. PRRSV-infected cell doublet (arrow) in a colchicine-treated culture; higher magnification, 50 μm; B. Control – higher magnification, 50 μm; C. Arrow indicates PRRSV-infected cell doublet; green-only (compare to red + green in A), 50 μm; D. Arrow indicates position of PRRSV-infected cell doublet, red-only (compare to red + green in A), 50 μm; E. Emerging clusters at 41 h p.i., 20 μm; F. Cytochalasin D treatment (1 μM) at 18 h p.i., 20 μm; G. control 42 h p.i. with PRRSV (arrows = actin fibrils; 10 μm). (Length of scale bar is indicated for each panel)

During the logarithmic phase of viral replication (e.g. 42–48 h p.i.), infection was present mainly in clusters containing multiple PRRSV-positive MARC-145 cells, routinely observed against a PRRSV-negative background of confluent cells (examples indicated by arrows in Figures 3B,C,D; see also Figures 5C,D and 6B). These clusters were never observed in the primary infection analyses, although occasionally infected cell doublets were seen at 20–22 h p.i.. Based on >10 independent analyses, we estimate that about 90% of secondary PRRSV infection of MARC-145 cells line is characterized by formation of infected cell clusters.

**Figure 8:**
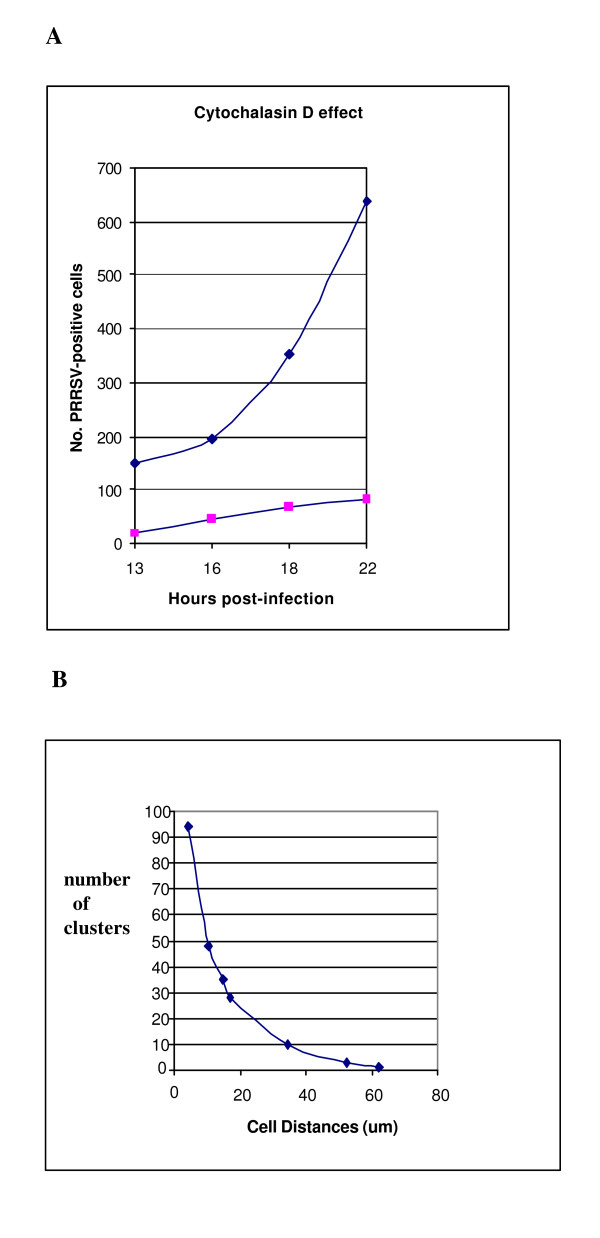
Time-course of PRRSV infection in cytochalasin D-treated (1 μm pretreatment) and control MARC-145 cells (A), and relationship of cell-to-cell distance and formation of secondary PRRSV-infected cell clusters (B).

Typically, dozens of clusters were seen in each culture, often containing a relatively central, bright-staining cell (e.g. Figure 3B,D), suggesting that cell-to-cell spread originated from a single PRRSV-infected reservoir cell. Absence of cluster formation during primary infection did not appear to be due to insufficient M.O.I. or late-stage development of a soluble cluster-inducer during culture, since using up to about 100-times the standard virus dose (obtained from 45–96 h p.i. cell supernatants) did not induce cluster formation at 20–22 h p.i. in our experimental system (data not shown), and cluster formation was also density-dependent (see below – Figure 8B). Clusters of PRRSV-positive cells were maintained for up to 72 h p.i. as illustrated in Figure 3D, and images at this time were also suggestive of maintenance of central bright-staining cells as reservoirs of virus.

### Role of the cytoskeleton in PRRSV infection

Treatment of cells with 10 μM colchicine simultaneously with PRRSV inoculation, resulted in about 75% inhibition of secondary PRRSV infection (e.g. 70% in control vs. 18% in colchicine-treated culture; counting >1000 total cells at 46 h p.i.) and data representative of numerous experiments are shown in Figure 5E to illustrate the inhibitory effect of colchicine on formation of PRRSV-infected secondary clusters, which are seen spreading throughout the controls (Figure 5C,D). Similarly, exposure of cells to cytochalasin D under several experimental conditions (1 or 2 μM; administered either simultaneously with virus infection or 2 h pre-infection) inhibited PRRSV-positive cells by up to 87% (illustrated in Figure 8A between 13–22 h p.i.; 2 h pre-treatment with 1 μM). These drug effects were confirmed by flow cytometric analyses (about 50–90% inhibition of PRRSV-positive cells; data not shown).

**Figure 7:**
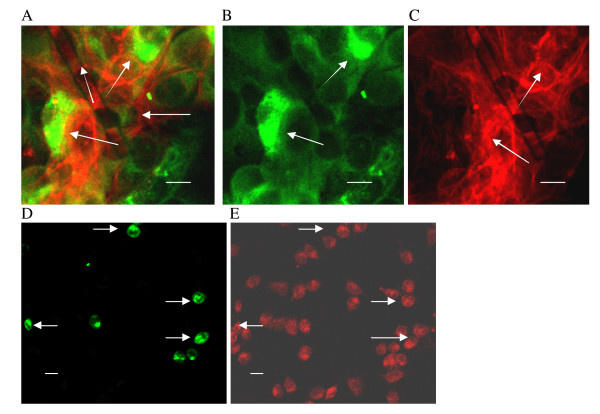
**Two-color fluorescence detection of actin (red) and PRRSV antigen (green) in MARC-145 cells**. A-C. Control 41 h p.i.: A = actin (red) + PRRSV Ag (green); B = PRRSV Ag only; C = actin only; arrows = actin fibrils surrounding uninfected cells, 25 μm; D (LDV antigen) & E (actin) expression in LDV-infected primary mouse macrophages; arrows indicate LDV-infected cells; 10 μm. (Length of scale bar is indicated for each panel).

To further evaluate PRRSV transmission *in vitro*, actin expression was imaged using Alexa Fluor 594-phalloidin (red). Similar patterns of actin expression were observed in control uninfected (Figure 5A) and PRRSV-infected (Figure 5B) cells, and all cells expressed some degree of actin when high levels of gain were applied. Cells often displayed fibrillar actin extensions appearing along the outer membrane as well as less-elongated extensions over the body of the cell. Effects on the actin staining pattern were apparent after exposure of MARC-145 cells to the actin disruptor cytochalasin D (Figure 6F), and also to some extent after exposure to the microtubule inhibitor colchicine (Figure 5E) although intact actin fibrils were still present (see also description of Figure 6A,C,D below). Simultaneous determination of actin expression and PRRSV infection demonstrated that relatively high expression of actin filaments correlated with PRRSV resistance, which was a consistent finding in dozens of control- and colchicine-treated experiments. This observation is illustrated in Figure 6A,C, &6D, where the arrows indicate the location of a PRRSV-positive doublet with low actin expression. Figures 5B and 6E also illustrate the negative correlation between actin and PRRSV-antigen expression. While this was not an absolute correlation (some PRRSV-positive cells did express high levels of actin), the trend was clear from >5 independent experiments. Actin fibrils also appeared to partition PRRSV-positive from PRRSV-negative cells and were often observed surrounding PRRSV-negative cells (Figures 6G and 7A–C; and see below). Cytochalasin D added at 18 h p.i. also suppressed secondary PRRSV-positive cluster formation (Figure 6F; 41 h p.i.; see emerging clusters in the control for this experiment Figure 6E), demonstrating a role for the cytoskeleton in cell-to-cell transmission.

The confluent cultures used in our experiments generally had mean cell-to-cell distances of < 2 μm, and the cells were usually in contact or too close to measure meaningful distances. Due to the wide dynamic range of the fluorescence signals, this is not always apparent in the figures, but some figures, such as 3A, 7A &7B, serve to illustrate the background of cultured cells. By seeding plates with different numbers of cells and then infecting these cultures containing different cell densities, it was possible to measure cell-to-cell distances in hundreds of cells per culture from the captured images, and then correlate the mean with the number of PRRSV-positive clusters (Figure 8B). The results show that formation of PRRSV-positive secondary clusters was density-dependent, with a direct correlation between the cell-to-cell mean distance and the number of clusters (Figure 8B).

In contrast to the ability of PRRSV to spread via cell-to-cell transmission, LDV did not exhibit this property, nor was there any dissociation of actin expression by primary cultured mouse macrophages from LDV permissiveness (probed during the replication peak, Figure 7D &7E). Also in contrast to the response of PRRSV in MARC-145 cells, treatment of mouse macrophages with the same concentrations of colchicine had little effect on LDV replication (+17%, -26%, and -21% LDV-positive cells relative to control in three separate experiments), although LDV infection was completely suppressed by cytochalsin D (no LDV-positive cells detected in three separate experiments).

Combined, these data show that after primary acute PRRSV infection of a small subpopulation of PRRSV-permissive MARC-145 cells, the virus spreads secondarily over the next 2–3 days to surrounding cells by cell-to-cell transmission. These virus mechanisms are both actin- and tubulin-dependent, demonstrating the critical role of the cytoskeleton in the processes of PRRSV infection and spread. In contrast, the related arterivirus LDV displays no cell-to-cell transmission in primary culture mouse macrophages and primary LDV replication is not tubulin-dependent as revealed by absence of colchicine sensitivity.

### Suppression of PRRSV infection by targeting viral permissivness with AK-2 and IFN-γ

**Figure 9:**
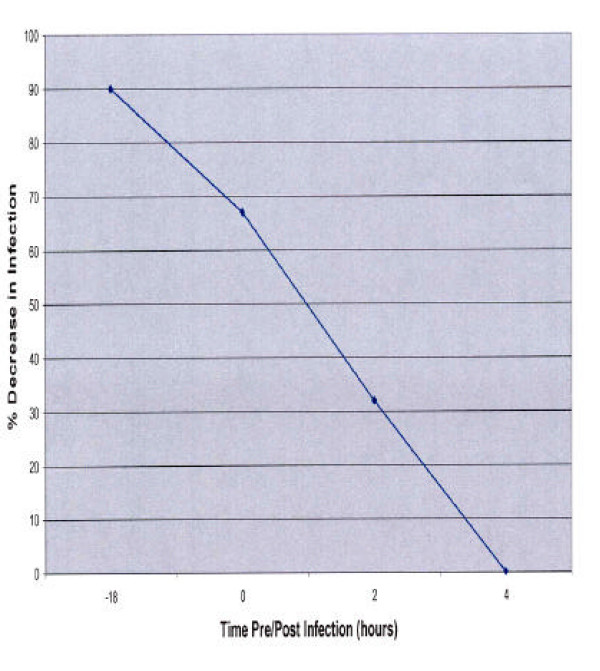
**Pharmacodynamics of PRRSV inhibition by AK-2**. MARC-145 cells were exposed to AK-2 at various times pre- or post-infection. The % inhibition of PRRSV antigen detection is shown on the y-axis.

Pretreatment of MARC-145 cells for about 18 h prior to PRRSV inoculation with AK-2 suppressed primary (Figure 2B) and secondary (Figure 2D) virus infection as assessed by flow cytometry. The pharmacodynamics of AK-2 inhibition of primary PRRSV infection were determined by microscopic FA analyses (Figure 9), demonstrating that the antiviral effect of AK-2 is on the PRRSV-permissive state rather than directly on the virus, since pretreatment was required to fully establish PRRSV resistance. No detectable morphological effects of AK-2 on MARC-145 cells were noted in our studies, and the PI profiles of treated cells were similar to those of control cells demonstrating intact and metabolically viable cells at 22–46 h p.i. (data not shown). AK-2 pretreatment completely inhibited the formation of secondary clusters of PRRSV infection, and only occasionally were single-positive cells observed at 42–46 h p.i. in AK-2-pretreated MARC-145 cells (Figures 3E &4C). However, when added at 20 h p.i., AK-2 only partially inhibited secondary (cluster) PRRSV spread, although there was a shift to single-cell PRRSV infection (Figure 3F). Partial inhibition of secondary PRRSV infection by delayed AK-2 addition was also observed by flow cytometry, since AK-2 added at 18 h p.i. inhibited the 42 h p.i. expression of PRRSV antigen, by about one-half (27% vs. 58% in the drug-vehicle control; Figure 2F). Exposure of MARC-145 cells to AK-2 simultaneously with PRRSV inoculation suppressed all secondary cluster formation at 45 h p.i. (see control in Figure 4E and AK-2-treated in Figure 4F), confirming that, even without pretreatment, AK-2 can suppress the viral permissiveness of target cells for secondary cell-to-cell transmission, while leaving primary single-cell infection relatively intact due to the time required for induction of PRRSV resistance.

A higher-magnification image of AK-2-treated (single-cell infection only) is shown in Figure 5F, which also illustrates the often-observed appearance of actin fibrils surrounding a PRRSV-positive cell, as if to separate it from other uninfected cells as indicated above for Figure 6G. The data also show the absence of a detectable effect of AK-2 on actin expression.

In other studies, AK-2 pretreatment inhibited PRRSV antigen expression at 20–22 h p.i. by about 90% in primary pig macrophages, in each of two experiments. Similarly, pretreatment of primary mouse macrophages with recombinant murine AK-2 inhibited LDV replication by 86% in a single experiment. Thus, the anti-arterivirus effects of AK-2 are expressed over a broad host cell range.

Observations were also made for the anti-PRRSV effect of IFN-γ pretreatment, which was demonstrated in previous studies to inhibit PRRSV replication [14]. PRRSV inhibition by IFN-γ was less effective than that of AK-2 under the same experimental conditions, as seen for example in the primary response to pretreatment measured by flow cytometry (Figure 2C,I); the effect of IFN-γ pretreatment also waned by 40 h p.i. (Figure 2J). FA analyses confirmed the reduced efficacy of IFN-γ relative to AK-2, since IFN-γ pretreatment did not completely inhibit formation of secondary PRRSV antigen-positive clusters (Figure 4A,D) and there were higher numbers of PRRSV antigen-positive cells in IFN-γ-pretreated cultures (data not shown). Consistent with these data, flow cytometry demonstrated that IFN-γ added at 18 h p.i. had a reduced effect on secondary PRRSV infection (Figure 2G), relative to AK-2 (Figure 2F) or the combination of AK2 and IFN-γ (Figure 2H); the inhibitory effect of post-treatment with both drugs is also shown in Figure 2H, although little if any synergy was observed. Thus, while IFN-γ mediated significant inhibition of PRRSV infection, AK-2 appears to be a more potent anti-PRRSV agent.

## Discussion

The results of this study show that PRRSV replication in an experimental MARC-145 cell system is composed of two discrete phases: primary infection of a relatively small subpopulation of PRRSV-permissive cells during about the first 22 h p.i, followed by secondary cell-to-cell spread over the next several days to contiguous cells, resulting in formation of infected cell clusters and ultimately cell death/CPE by days 3–4 p.i. Flow cytometry of PRRSV infection of pig macrophages has previously been reported [32], but for the present study we developed a flow technique to quantitatively measure PRRSV antigen expression in MARC-145 cells, a standard cell line for the study of PRRSV infection. Combined with FA analyses by microscopy, this methodology provides evidence that permissiveness to PRRSV infection is dependent on the mechanism of virus presentation, since the majority of cells appear non-permissive to free virus, but become readily infected by exposure to productively-infected cells.

These dynamics of PRRSV infection of MARC-145 cells stand in stark contrast to those of the related and relatively benign arterivirus LDV, since primary LDV infection of cultured mouse macrophages peaks at about 8 h p.i. *in vitro*, and soon thereafter *in vivo*, but there is little or no secondary virus replication after these events [17]. A likely explanation for this difference is the absence of cell-to-cell (cluster) spread of LDV. The ability of PRRSV to spread secondarily by cell-to-cell transmission may overcome an early block to virus permissiveness, and while it is not yet known whether this mechanism occurs *in vivo*, it could potentially help PRRSV resist antibody defenses and maintain persistence.

Our studies show that PRRSV transmission to infected cell clusters is dependent upon cytoskeletal function, since the microtubule inhibitor colchine [26-28] as well as the actin inhibitor cytochalasin D [26,29-31] suppressed secondary virus spread. Consistent with this conclusion, a number of other viruses are dependent on the host cytoskeleton for entry, transport, and/or egress [32-34], and actin polymerization may enhance cell-to-cell virus spread [35]. For example, the actin cytoskeleton is a critical factor for assembly and/or budding of HIV-1 [36], West Nile virus [37], respiratory syncytial virus [31], fowlpox virus [38], and equine infectious anemia virus [39]. Interestingly, we observed that the pattern of actin expression correlated with PRRSV resistance, since there was often a distinct appearance of actin filaments surrounding PRRSV-negative cells, and a general (but not absolute) negative correlation between viral antigen detection and the level of actin expression. This finding appears to suggest that actin provides a protective barrier to cell-to-cell transmission, and that the actin cytoskeleton may have a dual role in PRRSV infection. This seemingly paradoxical observation may be analogous to that reported for transport of secretory granules, which is both limited and mediated by the actin cortex [40]. Furthermore, the actin cytoskeleton is a potential barrier to exocytosis [41,42], and the cortical actin network may provide a cellular barrier to SFV [43] and HIV [29]. PRRSV infection might potentially inhibit the cytoskeleton [44-46], to further promote virus spread in culture. In contrast to the finding with PRRSV, there was no difference between actin expression in LDV-permissive and LDV-non-permissive mouse macrophages. Additional studies of the role of the cytoskeleton should be of interest to PRRSV pathogenesis and the biology of arteriviruses.

Primary infection was dependent on an intact cytoskeleton, and nascent cluster initiation during this time frame was signified by the occasional appearance of infected cell doublets. Formation of secondary PRRSV-infected cell clusters was a function of time p.i. and the cell-to-cell distance, and is thus a physical property of the *in vitro *system, potentially analogous to tissue sites *in vivo*. The viral dynamics from our studies are consistent with previous observations demonstrating infection of a small percentage of cells by day 1 p.i., which increases markedly over the next few days, culminating in peak supernant virus titers at about 72–96 h p.i. [9]. The data also suggest that 1 TCID_50 _contains multiple virions since the number of cells acutely infected can exceed the TCID_50 _dose and optimal infection is achieved at low M.O.I. (calculated by TCID_50_) as previously reported (9,14). Future studies to clarify the relationship of M.O.I and TCID_50 _might help to determine what special characteristics facilitate primary permissiveness to free virus, which could include ability to bind one or more virions as well as biochemical factors regulating virus replication.

Our data show that the logarithmic increase in the percentage of PRRSV infected cells over about 2–4 days p.i. is due to secondary cell-to-cell virus spread, from innately-permissive (reservoir) cells to surrounding uninfected cells. The foci of infection typically observed microscopically, in cultures of PRRSV-infected cells which begin to degenerate by 3–4 days p.i., are thus the outcome of secondary cluster infection and direct virus infection. These data reinforce that secondary spread to clusters in MARC-145 cells provides an important direction for future studies of PRRSV mechanisms, since cell-to-cell virus transmission [47] might help to explain the resistance of PRRSV to antibody-mediated control as well as PRRS pathology. In a recent study, the replication of PRRSV in transformed pig peripheral blood monocytes was shown to be under genetic control and varied between 23.1–31.4% at 24 h p.i. [48]. Thus, our results suggest the possibility that variations in cell-to-cell PRRSV transmission may underlie differences in PRRSV replication between different cell lines *in vitro*.

The present results show that AK-2 is a potent inhibitor of arterivirus (PRRSV and LDV) replication. This is the first published report of the antiviral effects of AK-2, which suppresses viral permissiveness by activating an antiviral gene program (Wong; unpublished), and which we exploited to supplement our studies of the IFN-γ response. Pretreatment was required for full expression of the drug effects, likely due to a lag phase for activation of the antiviral gene program. Both primary as well as secondary (cluster) PRRSV infection were susceptible to the antiviral actions of AK-2, but required optimal conditions of pretreatment for induction of the PRRSV-resistant state, and secondary PRRSV infection was controlled independently of primary infection by simultaneous or delayed drug exposure. IFN-γ was relatively less effective under our experimental conditions, but our IFN-γ data reinforce the conclusion that the viral-permissive state is an important drug target in PRRSV infection. This is also the case for LDV-mediated fetal infection [49,50] and neuropathology [51], since suppression of LDV-permissiveness by IFN-γ reduces these viral phenomena, and the arteriviruses may be good models for the role of permissiveness in antiviral strategies. Despite sensitivity to IFN-α, PRRSV may be a relatively weak inducer of this cytokine [52], facilitating evasion of host defenses. Thus, development of useful drugs which target viral permissiveness could be a superior strategy to inhibit primary or secondary phases of PRRSV infection, particularly if secondary cell-to-cell spread is resistant to a conventional antibody attack, and might also provide a superior toxicity profile, since the induction of PRRSV resistance is fundamentally a physiological process.

## Conclusion

PRRSV infection has been shown to spread by cell-to-cell transmission in a stable MARC-145 cell line. Two stages of viral infection have been identified: primary (innate) permissiveness to free-virus which appears in a relatively small percentage of cells, and secondary permissiveness to cell-to-cell transmission which is highly expressed and culminates in CPE. PRRSV infection of MARC-145 cells requires an intact cytoskeleton, but actin expression may also correlate with cell protection. Drugs such as AK-2 which induce a block in PRRSV permissiveness reveal a potentially important drug target for suppression of primary and secondary PRRSV infection.

## Methods

### MARC-145 cells

A stable and mycoplasma-free MARC-145 cell line was utilized in these experiments. Cells were cultured in DMEM containing10% fetal bovine serum, and for virus infections the medium was switched to MEM containing 2% horse serum. Cells for virus infections were grown to confluency in either T-25 flasks (seeded with about 5 × 10^5 ^cells/culture) or 8-well glass slide chambers (seeded with about 10,000 cells/culture; Lab-Tek II; Nalge Nunc International), and for the cell density studies, serial two-fold dilutions of the cells were used. Cells were inoculated with PRRSV at about 1–2 days after seeding (time to approximate doubling of the population).

### Primary pig macrophages

Pig cells were collected from 4–8 week old pigs by lung lavage with PBS [21-23]. Cells were cultured in DMEM containing 10% FBS. After 18–24 h, non-adherent cells were removed by washing. The remaining adherent cells were cultured for an additional 24 h in RPMI containing 2% horse serum, and then inoculated with PRRSV (M.O.I. approximately 0.1 TCID_50_).

### PRRSV stocks

PRRSV isolate SD-23983 was passaged on MARC-145 cells, preparing high-titer (~ 10^5 ^TCID_50 _per ml) virus stocks from culture supernatants at 48–96 h p.i. PRRSV stocks were sequentially filtered through 0.45, 0.22, and 0.10 um filters and confirmed to be mycoplasma-free by testing on PPLO medium. As reported previously (9,14), maximum efficiency of PRRSV infection of MARC-145 cells occurs with low M.O.I as determined by TCID_50_, probably due to the presence of multiple virions per TCID_50_. For the present studies, M.O.I. of about 0.01 TCID_50 _(slide cultures) and 0.001 TCID_50 _(T-flask cultures) were found to result in near-optimal efficiency of infection, and were thus used for our studies unless otherwise noted in Results.

### Fluorescence (FA) detection of viral and cellular target molecules

PRRSV replication was detected using FITC-labeled IgG anti-PRRSV nucleocapsid monoclonal antibody (SDOW17; [24]). MARC-145 cells were cultured and inoculated with PRRSV in glass-bottom slide chambers, fixed in 80% acetone, and incubated for 1 h at 37°C with a 1:100 dilution of FITC-conjugated SDOW17 antibody made in PBS containing 5% fetal bovine serum. Then the cells were washed three times with cold PBS prior to examination under a fluorescence microscope, screening about 30–40,000 total cells to obtain the incidence of antigen-positive cells. Confocal fluorescence microscopy was performed using an Olympus BX61 microscope and Fluoview software. Images shown in Figures 3 and 4 display the yellow scale bar captured with the original image, along with a higher-contrast white scale line. For flow cytometry, MARC-145 cells were cultured and PRRSV-inoculated in T-flasks, the cells were suspended in trypsin-versene, pelleted at 1000 rpm, resuspended in DMEM with 2% horse serum, fixed in cold 80% acetone for 10 min, washed twice in PBS, and resuspended in 1 ml PBS containing 60 ul of fetal bovine serum. FITC-conjugated SDOW17 antibody was then added to the cells (2.5 ul/ml), incubation was carried out at 37°C for 60 min, the cells were washed with PBS, examined under a fluorescence microscope, and flow cytometry was performed with a FACSVantage SE (Becton Dickenson) equipped with a 488 Enterprise II coherent laser. Twenty thousand events per sample were analyzed with CellQuest software. Cell cycle analyses were also performed on the same samples, by staining with propidium iodide for 20 minutes at room temperature and analyzing the flow cytometric results with ModFit LT 2.0 software. PRRSV replication in primary pig alveolar macrophages [22,23] was assessed by FA under a fluorescent microscope. Cellular expression of actin was determined by incubating acetone-fixed cells with AlexaFluor 594 phalloidin (Invitrogen) according to the manufacturer's instructions, with the modification of simultaneous PRRSV detection as above, such that combined labels were applied for 60 min at 37°C, permitting two-color fluorescence detection by confocal microscopy. Data shown are representative of at least 2 replicate experiments for each type of experiment described in the Results.

### LDV infection of primary mouse macrophages

Peritoneal macrophages were collected from outbred ICR mice, seeded onto glass coverslips, and inoculated with a standard dose of LDV-P as described previously [25]. LDV replication was assessed in cells fixed in acetone at 8 h p.i by IFA assay as described previously [25].

### Drug treatments

Purified recombinant human interferon-γ (IFN-γ; 100 ug/ml) and actokine-2 (AK-2; 50 ug/ml) were provided by Actokine Therapeutics. AK-2 is a cytokine-based experimental antiviral being developed by Actokine Therapeutics, which consists of recombinant normal human proteins comprising part of the mammalian cell response to virus infection (Wong; unpublished). Soluble stocks of these agents were stored at 4°C in fetal bovine serum, which also served as the drug-vehicle control for the experiments, and were diluted 1:50 or 1:100 in medium to yield concentrations in cell cultures of about 1–2 ug/ml. As noted for individual experiments, cells were exposed to the drug or control treatments prior to PRRSV infection (pretreatment), during the course of PRRSV infection (delayed or post-treatment), or simultaneously with PRRSV infection. Based on previous studies of *in vitro *efficacy, the microtubule inhibitor colchicine which binds to tubulin (Sigma; 5 or 10 μM; 26–28) or the microfilament disruptor cytochalasin D which depolymerizes actin (Sigma; 1 or 2 μM; [26,29-31]) were added to cell cultures at the times indicated.

## Competing interests

The author(s) declare that they have no competing interests.

## Authors' contributions

WAC conceived and designed the study, carried out experiments, performed data collection and analyses, and drafted the manuscript. RGD was responsible for cell culture, carried out some of the fluorescence analyses, and contributed to the FACS analyses. GHW prepared AK-2, IFN-γ, and control reagents and contributed to the experimental design. SS performed the FACS analyses. PWD carried out infection assays and performed some of the manual microscopic analyses. RRRR prepared pig macrophages, MARC-145 cells, and PRRSV for the project. EAN prepared the antibody reagent, MARC-145 cells, and PRRSV for the project. All authors made intellectual contributions to the study, and participated in the review and revision of the manuscript.
